# Microstructure and Soil Wear Resistance of D517 Coating Deposited by Electric Spark Deposition

**DOI:** 10.3390/ma14205932

**Published:** 2021-10-09

**Authors:** Min Wei, Qiang Wan, Shanjun Li, Liang Meng, Daocheng Cao, Chaoyue Dai, Yongjun Huang, Yangyi Xiao, Wanjing Dong, Kan Zheng

**Affiliations:** 1 College of Engineering, Huazhong Agricultural University, Citrus Mechanization Research Base, Ministry of Agriculture and Rural Affairs, Wuhan 430070, China; weimin_un@163.com (M.W.); Meangliang2006@mail.hzau.edu.cn (L.M.); danyuecanxin@163.com (D.C.); huangyj@mail.hzau.edu.cn (Y.H.); yyxiao@hzau.edu.cn (Y.X.); dwj@mail.hzau.edu.cn (W.D.); zhengkan0219@163.com (K.Z.); 2 Sunny Group Co., Ltd., Ningbo 315000, China; Daichaoyue@mail.hzau.edu.cn

**Keywords:** D517 coating, pulse current, soil wear, adhesion

## Abstract

The abrasion failure is the key factor for prolonging the service life and energy saving of furrow openers. The hardness enhancement was reported to be an effective strategy to increase the wear resistance against the soil abrasion. D517 coatings were deposited on Q235 steel by electric spark to improve the wear-resistant property with an affordable cost for farmers. The wear behavior of the coatings was characterized in a pin on disk friction equipment and a homemade soil abrasion simulation system. The soil adhesion, which is highly related to energy consumption, was also evaluated. Results showed that D517 coatings revealed dendrite structure with some randomly distributed carbides. The electric current exerted a great influence on the microstructure, hardness, friction coefficient, and soil wear rate. The wear rate of samples deposited with 80 A and 90 A reduced to 79% and 84%, respectively, as compared with the normalized heat-treated 65 Mn steel after 6 h in soil. This work provides a promising solution to increase the wear resistance of furrow openers. It needs to be noted that the coating would increase the soil adhesion of the opener, which needs to be further explored to decrease the energy consumption.

## 1. Introduction

Soil operation is an important part of mechanized production in the agricultural field [1]. Soil abrasion can be induced by the large number of hard particles in sandy soil during cultivation [2,3]. The accumulation of soil abrasion would lead to passivated outer corners and reduce contour [4]. The wear rate is mainly influenced by soil mechanical conditions, the working surface structure of the parts, and the component materials of the agricultural parts [5]. Therefore, it would be helpful to obtain the wear regularity and understand the wear mechanism of the furrow openers in the soil for improving the wear resistance of furrow openers.

Many researchers have indicated that the wear resistance of components in a hard abrasive such as soil could be improved by increasing the hardness of contact surface [6]. Traditional heat treatment of quenching could increase the wear resistance but inevitably sacrifice the total toughness. Surface modification was reported as the prior method to improve the wear resistance because of its high hardness and lower cost as compared with the integral heat treatment. Surface modification mainly consists of surface heat treatment and cladding coating. Surface heat treatment such as plasma arc scanning and laser quenching was widely applied in 45 steel and 40Cr alloy steels, and higher hardness was achieved in the surface to decrease the wear rate [7,8]. However, the increment of hardness and wear resistance also were limited by the compositions of matrix materials. Coatings’ thickness, adhesion, and hardness should be properly considered as factors for the agriculture parts. Thus, coatings could be prepared by laser cladding, plasma cladding, and electrical spark. Bosio et al. prepared a WC coating using a metal sintering process and Bartkowski prepared Stellite-6/WC coating with laser cladding technology; they both obtained good wear resistance under simulated soil wear conditions [9,10]. These results suggested that the cladding coating is an effective method to increase the wear resistance. It needs to be noted that the reported plasma arc and laser technologies are prone to produce cracks for large-area surfaces due to their high-power density. Electrical spark, which is especially proper for farmers, possesses the characteristic of low cost.

At the same time, in the special working environment of the soil-touching parts of agricultural machinery such as furrow openers, the furrow openers need to have a hard surface and maintain sufficient internal toughness. If a harder wear-resistant coating can be prepared on a substrate with good toughness, it will undoubtedly solve the technical problem that the furrow opener has both a hard surface and good internal toughness. The difficulty is that, in the process of preparing a thicker wear-resistant coating, the preparation method has to have a small impact on the mechanical properties of the substrate. The electro-spark deposition technology is electrostatic discharge, where the electrode material migrates to the surface of the substrate at a high speed, the bonding is firm, and it has little effect on the structure and mechanical properties of the substrate. If the coating is partially damaged, it can be repaired by electro-spark deposition, which has high economic value. Electric spark deposition technology is considered as a promising technology in improving the surface wear resistance of agricultural machine in soil with tough components [11]. Penyashki et al. [12] studied the wear resistance of TiC, TiN-based cemented carbide electrode composite coatings of high-speed steel by non-contact local electrical spark deposition (LESD) technology. Results suggested that coated HS6-5-2 Steel presented 2.5 times the wear resistance as compared with the bare steel.

In addition, surface modification of furrow openers mainly focused on the hardness and friction coefficient, while the soil wear weight loss was seldom reported due to lack of wear-simulating equipment. Additionally, researchers in the past often focused their attention on how to improve their wear resistance in order to extend their service life, but ignored the adhesion of the parts after the coating strengthened. In this paper, financially feasible electric spark deposition was deposited on D517 coating on Q235 steel substrate against the soil wear to prolong the life of the furrow openers. The wear regularity was studied with a homemade trenching machine to simulate the service state. At present, no uniform standard has been established for the soil adhesion test, leading to lack of a commercial machine. Thus, the soil adhesion test was conducted by the self-made machine in this paper.

Most of the energy consumption of agricultural machinery comes from overcoming the resistance produced by soil tillage components. Soil adhesion greatly affects the energy consumption. According to the literature, soil adhesion increases the resistance of ploughing by more than 30% [13]. Reports suggested that the energy consumption of tillage machinery increases by 30% to 50% [14]. Therefore, determining the influence of the microstructure and hardness of the coatings on the parameters of wear and on the adhesion of the soil to the coating is an important aspect for the surface modification of the component that interacts with the soil. The results would provide scientific guidance for the study of furrow blade wear and surface modification.

## 2. Materials and Methods

### 2.1. Coating Preparation

D517 coatings were deposited on Q235 steel (the chemical composition is shown in Table 1) substrate by electric spark (YJHB-2 precision welding repair machine, Yijing Electronics Co., Ltd., Yuyao, China) using D517 welding wires with a diameter of 1.2 mm. The chemical compositions of D517 mainly consisted of Fe, Cr~10–16%, and C~0.25%. The high Cr content was expected to enhance the hardness by forming high-density chromium carbides and to provide excellent corrosion resistance against the moisture corrosion of the soil. The substrates were cut into 40 mm × 40 mm pieces by wire-cut electrical discharge machining (EDM). Before the deposition process, the surfaces of the substrates were ground and polished to a mirror-like surface to enhance the adhesion between coatings and substrates. After pre-experimental debugging, the pulse time was fixed at 50 ms in order to study the effect of current on the coating structure; the deposition currents were 50, 60, 70, 80, and 90 A. Argon gas was used to ensure the welding quality. To decrease the thermal stress during the deposition process, the welding process was applied on both sides in turn and the sand covering was used to slow down the cooling rate. In addition, the surface slag was removed by hammering, which could also reduce welding stress and avoid welding cracks. The deposited coatings were about 2 mm in thickness, which provided enough materials for soil wear and assured long service time. After deposition, coatings were ground to obtain a smooth surface. As references, 65 Mn steel with a size of 30 mm × 30 mm was normalized after being treated at 825 °C for the soil abrasive wear test.

### 2.2. Microstructure Characterization and Hardness Test

The prepared D517 coatings were cut into 10 mm × 5 mm pieces by wire electrical discharge machining (EDM). The surface and section of the coating were ground and polished with metallographic sandpaper to get a mirror-like surface. Then, the samples were etched with a 30% nitric acid solution. The X-ray diffraction (XRD) was used to characterize the phase composition of the coatings. The microstructure of the surface and cross section of the corroded coatings were observed with an optical microscope (OM) and a ZEISS SIGMA field emission scanning electron microscope (FE-SEM). The chemical compositions were analyzed by energy dispersive spectroscopy (EDS, EDAX genesis 7000 EDS system, Philadelphia, PA, USA). An HV-1000B microhardness tester (Hua Yin, HV-1000B, Lanzhou, China) was used to measure the hardness from the surface and the cross section of the coatings. The measure load was 0.981 N with a holding time of 15 s. The hardness measurement was conducted several times to obtain an average value.

### 2.3. The Friction Test and Accelerated Wear Simulation Test

**Figure 1:**
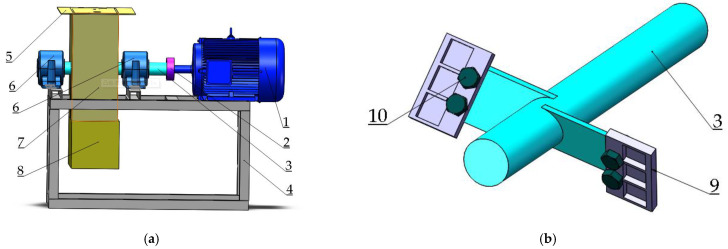
(**a**) three-dimensional diagram of a homemade soil wear simulation test bench, (**b**) sample holder. Notes: 1, three-phase asynchronous motor; 2, coupling; 3, main shaft; 4, support frame; 5, cover plate; 6 half-round seat ball bearing; 7, cover; 8, material box; 9, device for fixing the samples’ 10. bolt.

The friction coefficients were evaluated on the MS-T3000 ball-on-disk tester under room temperature and atmospheric pressure. Si_3_N_4_ balls with a diameter of 3 mm, which possess similar hardness to the hard particles in soil, were chosen as mated materials. The reciprocating sliding test was carried out with a load of 5 N; the sliding speed was 200 rpm and the total testing time was 15 min. Abrasive wear was reported to be the main mechanism for soil wear because of large amounts of sand and gravel [15]. To obtain the wear regularity in the laboratory, a homemade accelerated wear simulation tester was developed to simulate the soil abrasive wear of the opener during service. The schematic of a homemade accelerated wear simulation tester is shown in Figure 1. The tester mainly consisted of a motor (named 1 in Figure 1), a shaft (named 3 in Figure 1), and a specimen holder. The shaft was driven by the motor and equipped with the specimen holder. The holder was designed according to the commercial operator with a diameter of 300 mm and a helix angle of 30°, which was reported as the appropriate angle for a furrow operator [16]. A torque sensor was placed between the motor output shaft and the main shaft to record the torque, which could be used to judge if the samples were in the holder during the test. The cover (named 7 in Figure 1) was designed to protect the abrasives from flying, and a cover plate (named 5 in Figure 1) was reserved to add abrasives. The spindle speed was adjusted to 625 r/min by the inverter during the test based on the speed of the commercial operator. Corundum with a size of 24 mesh was selected as the abrasive due to its similar hardness as hard particles in soil. The time of the abrasive test was 6 h and the weight loss of the samples was measured every hour.

### 2.4. Soil Adhesion Test

**Figure 2:**
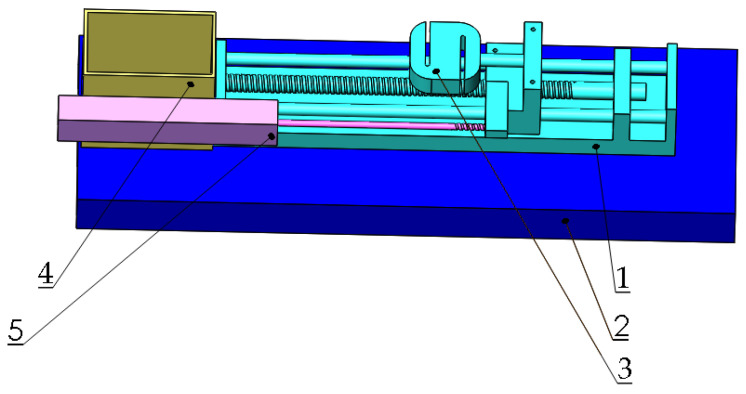
Three-dimensional diagram of the homemade soil adhesion test bench. Note: 1, biaxial ball screw sliding table; 2, base; 3, S-type tension and compression sensor; 4, container for soil; 5. KTC displacement sensor.

To get the adhesion properties of coated samples and the soil, a soil adhesion tester was designed, as shown in Figure 2. The device consisted of three parts. Samples were held in the shaft, which connected with a style tension and compression sensor. The shaft could be driven by the step motor. Additionally, the location of the sample during sliding was recorded by the KTC displacement sensor. During the test, the sample was buried into the soil, and the related drive force as a function of displacement was obtained in the screen. In order to ensure the accuracy of the results, each sample was tested in two modes: the single side adhesion test and total buried resistance test. The single side adhesion was tested by the soil friction coefficient meter (Xiamen Yishite Instruments Co., Ltd., ST-MXZ-1, Xiamen, China). The soil used for the test came from the agricultural experiment field on the campus of Huazhong Agricultural University. The soil type was silty clay [17]. To calculate the original moisture content of the soil, it was heated in a resistance furnace at 107 degrees Celsius for 8 h.

## 3. Results and Discussion

### 3.1. Microstructure of D517 Coatings

**Figure 3:**
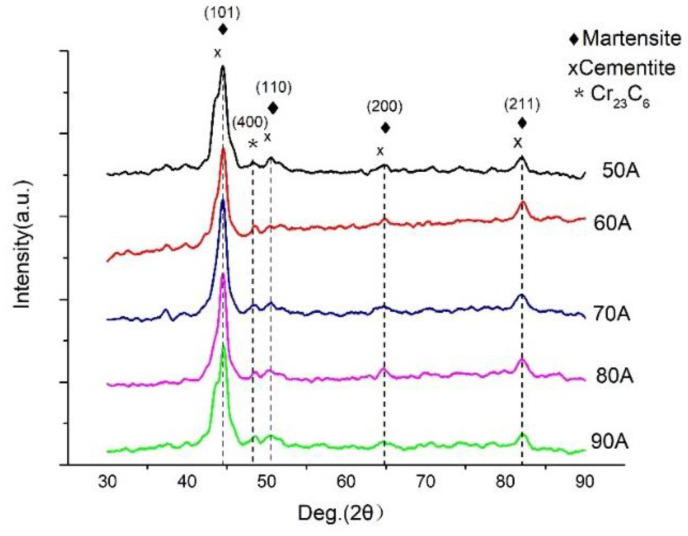
XRD patterns of D517 coatings deposited at different currents.

**Figure 4:**
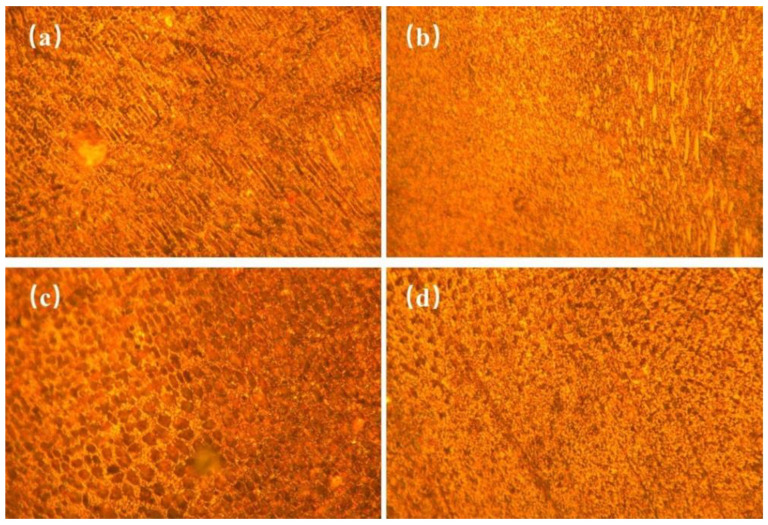
Microstructure of Deposition Layer at Different Currents: (**a**) 50 A, (**b**)70 A, (**c**) 80 A, (**d**) 90 A.

Figure 3 shows the X-ray diffraction patterns of Fe-Cr alloy coatings prepared under different currents. According to the XRD patterns, all samples displayed five characteristic peaks which were located at 44.4°, 48.2°, 50.5°, 64.8°, and 82.1°. Among them, the diffraction peaks at 44.4°, 50.5°, 64.8°, and 82.1° were related to the overlapping diffraction peaks of martensite and cementite, which corresponded to (101), (110), (200), and (211) crystal planes. Another peak located at 48.2° corresponded to the (400) crystal plane of Cr23C6. Figure 4 shows the microstructure of D517 coatings deposited with different currents. No defects such as cracks and voids were observed, which indicated that the coatings were well formed and the electro-spark deposition was suitable for the surface strength for the furrow openers. Combined with the X-ray diffraction results, the deposited coatings were typical dendritic structures composed of lath martensitic, cementite, and chromium iron carbide. As the current increased, the size of the primary carbide gradually increased and gradually become spheroidal carbide particles, as shown in Figure 4c. Further increasing the current, the spheroidal carbides disappeared. The coarsening of the lath martensitic structure was also observed and was assumed to be related to the increased input energy, which raised the temperature around the molten pool, resulting in an accelerated diffusion rate. The growth of the coating crystal structure was directly related to the diffusion rate of the elements. The faster the diffusion rate, the faster the tissue growth rate and the larger the shape. According to Fick’s first law [18]:

(1) J=dm/Adt=−D(dc/dx)

where *J* represents the diffusion flux, (the flow rate of the substance per unit area perpendicular to the diffusion direction *x* per unit time), *D* is the diffusion coefficient, and *dc*/*dx* is the concentration gradient. It can be seen that the diffusion flux is directly proportional to the diffusion coefficient. The diffusion coefficient [19]:

(2) D=Do exp(−Q/RT)

where *Do* is the frequency factor and *Q* is the activation energy of diffusion. Obviously, the activation energy of diffusion is directly proportional to the diffusion coefficient. According to the Welding Energy Input formula [20]:

(3) Q=IU/V

where *Q* is the Energy Input, *I* is the welding current (A), *U* is the arc voltage(V), and *V* is the welding speed (cm/s or mm/s). As the current increases, the input energy increases (the diffusion activation energy increases) and the diffusion coefficient increases. The final result leads to a larger diffusion flux. This is the main reason the crystal structure became larger as the current increased. In addition, the deposited coatings at 80–90 A (as shown in Figure 4c,d) showed a network and plate-like structure, which suggested that the current could also significantly affect the shape of the metallographic structure. A similar microstructure was also observed in the literature [21,22,23,24].

**Figure 5:**
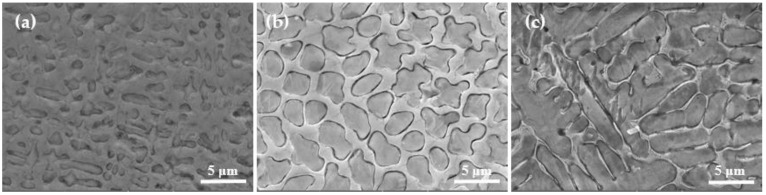
SEM images of D517 wear-resistant wire coating under different currents. (**a**) 50 A (**b**) 80 A (**c**) 90 A.

The SEM was employed to further observe the microstructure coarsening for samples deposited with 50 A, 80 A, and 90 A, as shown in Figure 5. For coatings deposited with 50 and 80 A (as shown in Figure 5a,b), the primary carbide revealed an oval shape with a size about 2.5 μm, and then translated into a strip shape with larger size, over 10 μm, as the current increased to 90 A (as shown in Figure 5c), which confirmed that the current could affect both the shape and size of carbides in D517 coating. It could be concluded that the excessive current leads to an increase in local high temperature and accelerates the diffusion rate of the element, resulting in a coarse crystal structure [25]. From Figure 5a–c, it can be observed that the grain boundary structure showed better resistance to a corrosive than an intracrystalline structure, which should be related to the higher Cr content in the grain boundary structure [26].

### 3.2. Chemical Composition Analysis

**Figure 6:**
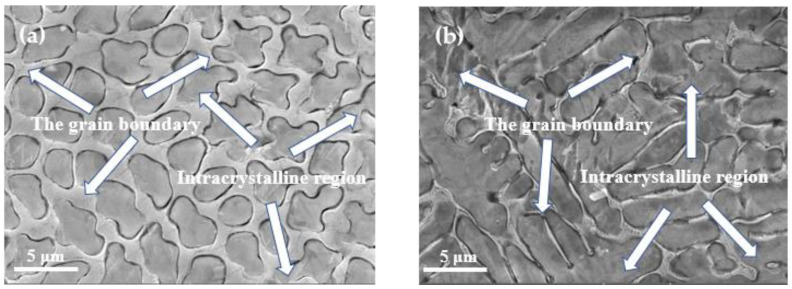
Typical microstructure of coatings deposited by 80 and 90 A. (**a**) 80 A (**b**) 90 A.

The chemical composition of coatings deposited at 50, 80, and 90 A was analyzed by EDS at a magnification of 10 k (as shown in Table 2). Results showed that D517 coatings were rich in Cr and Fe and poor in C, O, Si, and Mn. The observed high carbon content should be related to the pollution introduced during the sample preparation. With the increase of current, the proportion of the Fe element gradually increased and Cr decreased, which is speculated as being related to the molten matrix. During the deposition process, the molten matrix was mixed with the near coating, which brought a large amount of Fe into the molten pool and solidified in the coating after the temperature decreased [27]. The higher the current is, the higher content of Fe is. For this reason, the speed of EDM deposition needs to be properly controlled to eliminate the melting of the substrate. In addition, high Cr content in the D517 coating is expected to provide excellent corrosion resistance and present good performance during soil cultivation in paddy field operations due to the dense passive film. Additionally, the chemical composition of the grain boundary and intracrystalline region were studied. Table 3 reveals the composition difference between the grain boundary structure and intracrystalline structure. It suggests that the grain boundary structure presents higher content in Cr and less in Fe. The higher Cr and C in the grain boundary would result in higher hardness. Thus, the microstructure of the coating is a network of composite structure with the hard grain boundary phase surrounded by the soft phase. As the temperature increased, the grain boundaries became thinner (as shown in Figure 6a,b). The M7C3 compound is usually covered by other phases, so it is almost unrecognizable under an optical microscope [28]. The related literature shows that interconnected M7C3 carbides are embedded in the martensite structure [29]. Therefore, the elements of the polygonal structure were detected by SEM and EDS, as shown in Figure 6a,b. Combined with EDS analysis, the primary carbide (Cr + Fe)/C atomic ratios in the 80 and 90 A coatings were calculated to be 73.99: 26 and 70.18: 29.82, which were substantially consistent with the atomic ratio of 7:3 = 70:30 in (Cr Fe)7C3. It indicates that the primary carbide structure should be M7C3 (M=Cr, Fe), which was also observed by XRD. These carbides would bring significant precipitation strength. Besides these observed carbides, primary carbide M7C3 could also be embedded in the martensite structure [29] and induce significant solid solution strength. After welding, the fast cool rate of the substrate would promise fine and uniformly distributed M7C3 particles, which was expected to improve the hardness and stability of the coating.

### 3.3. Microstructure of the Interface between D517 Coating and the Substrate

**Figure 7:**
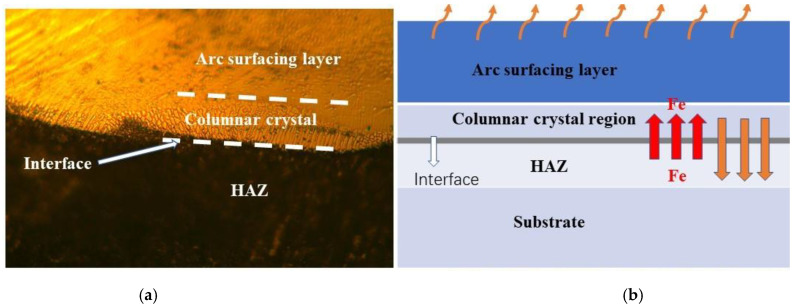
Microstructure near the interface of the deposition layer of the D517 wear-resistant welding wire. (**a**) Microstructure near the interface of cladding layer, (**b**) sketch map.

One of the most important requirements of hard coatings applied for improving wear resistance of a soil-engaging component is the excellent adhesion properties, which depend on the correlation of the coatings and the substrate. Thus, the interface between the substrate and D517 coatings was observed, as shown in Figure 7a. A transition zone between the arc surfacing layer and the substrate was formed and the zone was composed of a heat-affected zone (HAZ) and a columnar crystal zone from the substrate side to the coating, in turn. The formation of the transition zone should be related to the heat flux from the molten coating during coating preparation and the concentration gradient of the alloy elements. The formation of the transition zone is shown with a schematic diagram in Figure 7b. The deposition process could be divided into two stages: First is the heating stage during arcing and the second stage is the cooling process after the arc melted.

During the heating stage, the heat transfers from the molten pool to the substrate near the coating. The nearby substrate would be heated and lead to a phase transformation or slight coarsening. Additionally, the heating transfer would accelerate the mutual diffusion between the substrate and the coating and a lot of Fe would diffuse into the coating.

According to the first principal of Fick’s law, where *J* is the diffusion flux,

(4) J=−D dρ/dx

and *D* is the diffusion coefficient (its value increases with increasing temperature), *ρ* is the mass concentration of the diffused substance, and *dρ*/*dx* is the concentration gradient along the thickness direction [30]. For coatings in this work, the concentration gradient was the same and the diffusion flux depended on the diffusion rate. The diffusion rate *D* is always positively related to the temperature. As the current increases, the temperature of the interface increases and enhances the mutual diffusion. Therefore, an increased content of Fe was observed by EDS in coatings as the current increased (as shown in Table 2). It needs to be noted that the mutual diffusion between the coating and substrate led to a metallurgical bond between the coating and the matrix and resulted in excellent adhesion. In the second stage, the heating diffused through the coating from the substrate side to the top surface. This means the interface possessed the highest temperature and the top surface possessed the fastest cooling rate. Thus, a columnar crystalline region formed in the interface and the preferred orientation of the columnar structure was perpendicular to the interface and stretched to the top surface.

### 3.4. Microhardness of D517 Coatings

**Figure 8:**
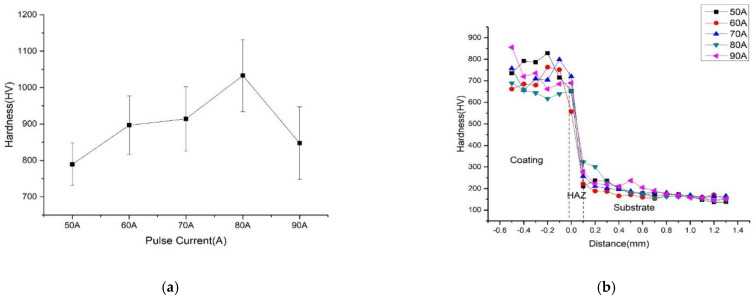
Surface and Section Hardness of D517 Coatings Deposited by Electric Discharge at Different Pulse Currents. (**a**) Surface Hardness of D517 Coatings with Different Currents. (**b**) Hardness of D517 Coatings to substrate at Different Currents.

Figure 8a shows the surface hardness of the D517 coating. The hardness firstly increased with the current and then decreased. The maximum hardness was achieved at the sample deposited with 80 A. As described by the literature, the hardness of the coatings prepared by the Fe-Cr-C compound depended on the number of carbides and the grain size [31,32]. Under 50–80 A, the hardness was mainly affected by the number of carbides because the heat generated by the current was not enough to complete the formation of carbide in the coatings. As the current increased, more carbides formed in the coating and enhanced the dispersion strengthening, which greatly improved the hardness [27]. When the current increased to 80 A, the carbides were totally formed and the grain size was limited to 2.5 μm and resulted in the maximum hardness. With a further increase in current to 90 A, the grain size of the coating coarsened to 10 μm and led to a significant decrease in hardness based on the Hall–Patch formula [33].

Figure 8b shows cross-sectional hardness of the layer from the top surface of the coating to the substrate. All samples revealed a similar trend in hardness variation. According to the hardness variation, the samples can be divided into high microhardness zone, transition zone, and low microhardness zone, which correspond to the coating, heat affected zone, and substrate, respectively. The thickness of the D517 coating was about 0.8 mm and 0.2 mm for HAZ. The slight variation in hardness of the coating was related to the microstructure heterogeneity caused by the dendritic segregation and nonuniform distribution of carbides. From the top coating to the transition layer, the hardness greatly decreased and quickly reached to the level of the substrate, which indicated the toughness of the substrate could be retained.

### 3.5. Surface Friction and Abrasive Wear

**Figure 9:**
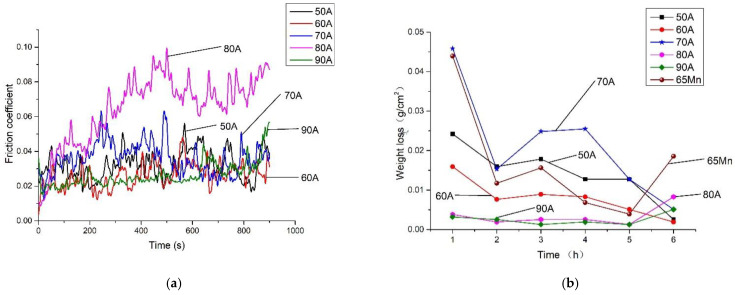
Surface friction and abrasive wear of D517 under different currents. (**a**) Surface friction. (**b**) Abrasive wear.

Figure 9a shows the surface friction coefficients of the D517 coatings deposited with different currents. All coatings revealed relatively lower friction coefficients under 0.1, which should be related to the large hardness and smooth surface [34]. From the friction coefficient curves functioned with the time, the friction coefficient gradually increased, then decrease, and finally stabilized to a certain range. The increase in the friction coefficient was due to the debris generated by the wear, which increased the scratch resistance during the sliding of the pin [35]. When the pin totally contacted with the samples, the coefficient became stable. In the range of 50–70 A, the friction coefficient increased with the current. The coating friction coefficient of coatings deposited by 80 A was the highest because of the increased roughness brought by the large number of precipitated carbides.

Figure 9b shows the weight loss of the D517 coatings as a function of the test time under the accelerated wear simulation tests. Additionally, the normalized 65 Mn was tested as reference. The samples were subjected to abrasion tests in 24 mesh corundum for 6 h and the abrasion was recorded every hour. The classic theory of wear generally regards hardness as an indicator of wear resistance [36]. The weight loss of coatings deposited by 50, 60, and 70 A firstly decreased and then increased, followed by a decrease. The initial large wear rates are related to the ground of the sharp corner of the square samples. The following increase was due to the relatively small hardness of the transition zone of the coating. After the totally worn out of the coating in the contact area, the contact area of the sample with the soil decreased and resulted in a lower wear rate. From Figure 9b, the coatings deposited with 80 and 90 A presented the lowest weight loss due to their high hardness. As the abrasive time increased to 5 h, the surfaces of the coatings deposited with 80–90 A were worn down and the internal transition zone possessed lower hardness exposure in the soil, resulting in an increase of weight loss during the sixth hour. For coatings deposited with a current under 80 A, the increase of the weight loss appeared at the third hour because of the relative lower hardness of the internal transition zone. As the test time further increased, the weight loss of the coatings deposited under 80 A slightly decreased. The decrease should be related to the shape change of the sample under abrasive wear. Like the agriculture parts, the cutting edge, which directly contacted with the soil, would be ground blunt to avoid the abrasive. Wear depends not only on the hardness, but also on contact areas, whose varied shape changes during wear [37].

In the process of wear, the failure of the coating was mainly attributed to the shedding of the carbides. When subjected to a large load, the surface layer deformed and cracks might be initiated at the interface between the metal and the carbides and extended. Abnormal wear of the coating deposited with 70 A conditions may be related to the shedding of carbides. The coating deposited by 90 A also revealed excellent wear resistance, though the hardness was much lower as compared with the coating deposited with 80 A.

According to the literature [31], the wear resistance of the coating is related to the carbide content in the coating. As the carbide content increases, the wear resistance is enhanced. D. Philippon et. al. [38] found that increasing the silicon content affects the structure and improves the mechanical properties and wear resistance. Secondly, increasing the Si content (and, thus, increasing the number of amorphous phases) can reduce the development of high residual compressive stress and, thus, obtain better performance. According to the EDS analysis, the coating deposited with 90 A revealed a high content of carbides and Si, which provided the observed low wear rate. Overall, the high-hardness D517 covered coatings deposited by EDM with a current over 70 A had a smaller amount of wear as compared with the referenced normalized 65 Mn steel. The total weight loss of samples deposited with 80 A and 90 A was 79.74% and 84.81% lower, respectively. It could be concluded that the D517 coating deposited by EDM is a good candidate to strengthen agricultural machine parts.

### 3.6. Soil Adhesion Test

**Figure 10:**
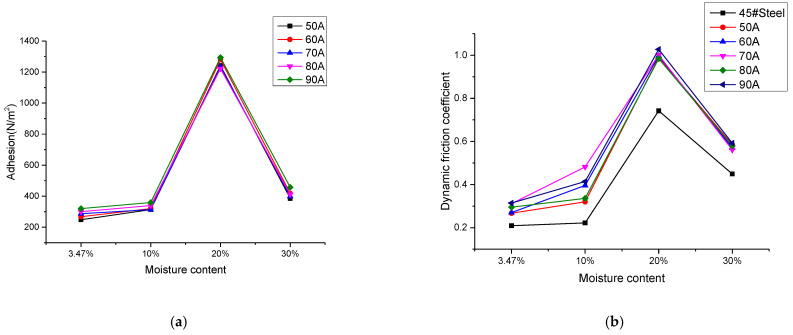
Adhesion and dynamic friction coefficient of coatings with different moisture content. (**a**) Adhesion. (**b**) Dynamic friction coefficient.

The adhesion force was tested with two different pieces of equipment. Figure 10a shows the adhesion of the D517 coating varying with the moisture content of the soil, as tested by the self-made tester. Figure 10b shows that the dynamic friction coefficient of the D517 coating varied with the moisture content of the soil. On the whole, the trends of the two graphs are the same, which verifies the accuracy of the self-made tester. The following conclusions were drawn. As the moisture content increased, the adhesion of the D517 coating soil increased, and after the moisture content threshold was exceeded, the adhesion decreased. The coefficient of dynamic friction showed the same trend and the coefficient of dynamic friction of the coating was all than that of 45# steel.

According to the soil adhesion mechanics [39], the soil adhesion *F_q_* can be used in the formula to indicate:

(5) $$F_{q}={f^{\prime }}_{m}\left(N_{m}+S\sum^{\;}N_{ni}\right)+S\sum^{\;}N_{nj}+F_{f}$$

where the *N_m_* is the gravity of the soil to the sample, *N_ni_* is the force of the normal direction adhesion, *N_nj_* is the tangential direction adhesion force, F_f_ is the viscous force between the moisture in the dimples and the soil water film, *f′_m_* is the proportional coefficient between normal force and tangential resistance, and *S* is the contact area between soil and metal surface. In this paper, D517 coating samples were cut into the same size; so, they have the same *N_m_* and *f′_m_*. Therefore, the main factors affecting the coating soil adhesion force were *N_ni_*, *N_nj_*, *F_f,_* and *S*.

The normal adhesion and the tangential adhesion were the main factors affecting the soil adhesion. The formula of tangential adhesion force *N_nj_* is as follows [39]:

(6) $$\sum^{\;}N_{nj}=N_{nn}+N_{nm}+\tau_{n}’$$

where *N_nn_* is the moisture film viscosity of soil, *N_nm_* is the capillary negative pressure suction, and *τ_n_*′ is the meniscus adhesion. The formula of normal adhesion force *N_ni_* is as follows [35]:

(7) $$\sum^{\;}N_{ni}=N_{nx}+N_{nt}+N_{nm}+N_{nw}+N_{nn}$$

where *N_nx_* is the physical and chemical adsorption, *N_nt_* is the soil adhesion, and *N_nw_* is the adhesion of soil particles on meniscus. The *N_nx_*, *N_nt,_* and *N_nw_* mainly depend on the properties of soil particles and soil moisture content. The samples were in the same tested environment, so the three values should be the same. Related literature [39] shows that when a continuous moisture film is formed on the interface between soil and solid matter, its adhesion is mainly caused by the viscosity of the moisture film. The greater the surface tension, the thinner the moisture film and the greater the adhesion. Additionally, some literature shows that the increase of soil moisture content will also increase the adhesion force by increasing the capillary suction of wet soil and the adhesion force of the meniscus [39,40]. Therefore, the soil adhesion force continues to increase with the increase of moisture content until the moisture content of the liquid limit reaches the liquid limit [39,40]. When the moisture reaches the liquid limit, if the moisture content is still increasing, the adhesion force will decrease gradually [41]. The reason should be attributed to the lubricant. That is why the observed adhesion decreased gradually when the moisture content was more than 20% in this paper.

Figure 10b shows that the dynamic friction coefficient of the coating was greater than that of the 45# steel with a smooth surface. This may be that the smooth surface was not easy to adhere to the soil. In recent years, some scholars have found [42] that the non-smooth surfaces of natural organisms have excellent viscosity reduction effects and the non-smooth surfaces prepared by biomimetic textures also have good viscosity reduction effects. However, in this paper, the surface of the sample was irregular, which resulted in a poor viscosity reduction effect.

## 4. Conclusions

The influence of currents on the microstructure, hardness, friction coefficient, and soil wear rate of D517 coatings were studied in this paper.

(1) D517 coatings revealed a dendritic structure with some carbides distributed. From the top surface to the substrate, the whole coating was composed of top coatings, columnar grain structure zone, and HAZ.
(2) As the current increased from 50 to 80 A, the carbide content increased, resulting in increased hardness in coatings, and a maximum hardness of 1032.9 HV was achieved at 80 A. A further increase in the current led to coarsening of the dendritic structure and decreasing the hardness of the coating.
(3) D517 coatings possess the friction coefficient lower than 0.1. The soil abrasive simulation revealed the excellent wear resistance of D517 coatings prepared by electric spark. Under the same wear conditions, the total wear rates of the samples deposited with 80 A and 90 A decreased by 79.74% and 84.81%, respectively, as compared with the normalized 65 Mn. Taking the wear rate and economy into consideration, the electric spark-prepared D517 coating could be a good candidate to protect furrow openers from wear.
(4) The adhesion test under different moisture contents showed that the adhesive force increased with the increase of the soil moisture content. When the liquid moisture content limit was exceeded, the adhesive force first decreased, thus forming a layer of soil between the metal surface and the soil–water-mixed liquid film as lubricant. Although the coating reduced the wear rate of the opener, the soil adhesion of the coating increased.

## Figures and Tables

**Table 1:** The chemical composition of Q235 steel (wt.%).

Elements	C	Si	Mn	P	S
	≤0.22	≤0.35	≤0.14	≤0.045	≤0.05

**Table 2:** Element concentration of coatings at different currents wt.%.

Element	C	O	Si	Cr	Fe	Mn
50 A	7.32	1.98	0.46	22.54	67.22	0.19
80 A	6.93	1.52	0.34	15.77	75.14	0.27
90 A	8.23	1.12	0.46	9.96	79.65	0.55

**Table 3:** The 80–90 A element concentrations at the grain boundary and intracrystalline microstructures wt%.

Current	Element	C	O	Si	Cr	Fe	Mn
80 A	The grain boundary	5.92	0.85	0.7	12.38	79.91	0.24
80 A	Intracrystalline region	7.44	1.85	0.17	17.47	72.76	0.29
90 A	The grain boundary	8.19	0.96	0.54	7.83	81.94	0.51
90 A	Intracrystalline region	8.3	1.43	0.31	14.21	75.08	0.66

## Data Availability

Data sharing is not applicable for this article.

## References

[1] Cucinotta F., Scappaticci L., Sfravara F., Morelli F., Mariani F., Varani M. (2019). On the morphology of the abrasive wear on ploughshares by means of 3D scanning. Biosyst. Eng..

[2] Ryabov V.V., Motovilina G.D., Khlusova E.I., Sidorov S.A., Khoroshenkov V.K. (2016). Study of thestructure of new wear-resistant steels for agricultural machinery components after operational tests. Metallurgist.

[3] Myalenko V.I. (2018). An abrasive wear forecasting method in the design of soil-cutting tools. J. Frict. Wear.

[4] Gomez V.A., de Macêdo M.C., Souza R.M., Scandian C. (2015). Effect of abrasive particle size distribution on the wear rate and wear mode in micro-scale abrasive wear tests. Wear.

[5] Tekeste M.Z., Balvanz L.R., Hatfield J.L., Ghorbani S. (2019). Discrete element modeling of cultivator sweep-to-soil interaction: Worn and hardened edges effects on soil-tool forces and soil flow. J. Terramech..

[6] Ali H., Rahsepar M., Hayatdavoudi H. (2018). Fabrication and characterisation of functionally graded Ni-P coatings with improved wear and corrosion resistance. Surf. Eng..

[7] Yan M., Zhu W.Z. (1997). Surface treatment of 45 steel by plasma-arc melting. Surf. Coat. Technol..

[8] Chen Z., Zhu Q., Wang J., Yun X., He B., Luo J. (2018). Behaviors of 40Cr steel treated by laser quenching on impact abrasive wear. Opt. Laser. Technol..

[9] Bosio F., Bassini E., Salazar C.G.O., Ugues D., Peila D. (2018). The influence of microstructure on abrasive wear resistance of selected cemented carbide grades operating as cutting tools in dry and foam conditioned soil. Wear.

[10] Bartkowski D., Bartkowska A. (2017). Wear resistance in the soil of stellite-6/wc coatings produced using laser cladding method. Int. J. Refract. Met. Hard. Mater..

[11] Młynarczyk P., Spadło S., Bartos J. (2018). Selected properties of electro-spark deposition on carbon steel using the Alloy 400 electrodes. IOP Conf. Ser. Mater. Sci. Eng..

[12] Penyashki T., Kostadinov G., Kandeva M. (2017). Examination of the wear of non-tungsten electro spark coatings on high speed steel. Agric. Eng..

[13] Ren L., Wang Y., Li J., Tong J. (1998). Flexible unsmoothed cuticles of soil animals and their characteristics of reducing adhesion and resistance. Chin. Sci. Bull..

[14] Qaisrani R., Jian-qiao L., Khan M.A., Iram R. (2010). Soil adhesion preventing mechanism of bionic bulldozing plates and mouldboard ploughs. Adv. Nat. Sci..

[15] Richardson R.C.D. (1967). The wear of metallic materials by soil—practical phenomena. J. Agric. Eng. Res..

[16] Darmora D.P., Pandey K.P. (1995). Evaluation of performance of furrow openers of combined seed and fertiliser drills. Soil. Tillage Res..

[17] Zhou H., Li D., Liu Z.Y., Li Z.Y., Luo S.C., Xia J.F. (2019). Simulation and Experiment of Spatial Distribution Effect after Straw Incorporation into Soil by Rotary Burial. Trans. Chin. Soc. Agric. Mach..

[18] Sun F., Chen X., Zhao Z. (2018). Anodic passivation on the recycling of cemented carbide scrap by selective electro-dissolution. Waste Manag..

[19] Bernardi F., Behar M., Santos J., Dyment F. (2005). Diffusion of al implanted into α-hf studied by means of the nuclear resonance technique. Appl. Phys. A.

[20] Tang X.H., Zhou Y., He Y.Y., Zhu G.F. (2003). Study on technology of laser welding of powder materials. Laser Technol..

[21] Lai H.H., Hsieh C.C., Lin C.M., Wu W. (2016). Characteristics of eutectic α (Cr, Fe) -(Cr, Fe)23C6 in the eutectic Fe-Cr-C hard facing alloy. Metall. Mater. Trans. A.

[22] Yang J., Tian J., Hao F., Dan T., Ren X., Yang Y. (2014). Microstructure and wear resistance of the hypereutectic Fe–Cr–C alloy hard facing metals with different La_2_O_3_ additives. Appl. Surf. Sci..

[23] Zhou Y.F., Yang Y.L., Jiang Y.W., Yang J., Ren X.J. (2012). Fe–24 wt.%Cr–4.1 wt.%C hard facing alloy: Microstructure and carbide refinement mechanisms with ceria additive. Mater. Charact..

[24] Katsich C., Badisch E., Roy M., Heath G.R., Franek F. (2009). Erosive wear of hard-faced Fe–Cr–C alloys at elevated temperature. Wear.

[25] Gietzelt T., Toth V., Weingaertner T. (2019). Impacts of layout, surface condition and alloying elements on diffusion welding of micro. process devices. Mater. Werkst..

[26] Stupnyts’kyi T.R., Student M.M., Pokhmurs’ka H.V., Hvozdets’kyi V.M. (2016). Optimization of the chromium content of powder wires of the Fe–Cr–C and Fe–Cr–B systems according to the corrosion resistance of electric-arc coatings. Mater. Sci..

[27] Efremenko V.G., Chabak Y.G., Lekatou A., Karantzalis A.E., Shimizu K., Fedun V.I., Azarkhov A.Y., Efremenko A.V. (2016). Pulsed plasma deposition of Fe-C-Cr-W coating on High-Cr-cast iron: Effect of layered morphology and heat treatment on the microstructure and hardness. Surf. Coat. Technol..

[28] Chang C.M., Lin C.M., Hsieh C.C., Chen J.H., Wu W.T. (2009). Micro-structural characteristics of Fe–40 wt%Cr–xC hardfacing alloys with [1.0–4.0 wt%] carbon content. J. Alloys Compd..

[29] Kiminami C.S., Bolfarini C., Botta F., Walter J. (2002). Formation of novel microstructures by spray deposition process. J. Metastable Nanocryst. Mater..

[30] Yang C., Li Z., Liu L., Ye F., Wu S. (2019). High temperature behavior of a diffusion barrier coating evolved from ZrO_2_ precursor layer. Surf. Coat. Technol..

[31] Chang C.M., Chen L.H., Lin C.M., Chen J.H., Fan C.M., Wu W.T. (2010). Microstructure and wear characteristics of hypereutectic Fe–Cr–C cladding with various carbon contents. Surf. Coat. Technol..

[32] Lin C.M., Chang C.M., Chen J.H., Hsieh C.C., Wu W.T. (2010). Microstructure and wear characteristics of high-carbon Cr-based alloy claddings formed by gas tungsten arc welding (gtaw). Surf. Coat. Technol..

[33] Nieh T.G., Wadsworth J. (1991). Hall-petch relation in nanocrystalline solids. Scr. Metall. Mater..

[34] Yamaguchi K., Sasaki C., Tsuboi R., Atherton M., Stolarski T., Sasaki S. (2014). Effect of surface roughness on friction behaviour of steel under boundary lubrication. Proc. Inst. Mech. Eng. Part. J-J. Eng. Tribol..

[35] Li C.D., Li B., Shen Y., Jin M., Xu J.J. (2018). Effect of surface chemical etching on the lubricated reciprocating wear of honed Al–Si alloy (Article). Proc. Inst. Mech. Eng. Part. J-J. Eng. Tribol..

[36] Archard J.F. (1953). Contact and rubbing of flat surfaces. J. Appl. Phys..

[37] Colaço R., Vilar R. (2003). Abrasive wear of metallic matrix reinforced materials. Wear.

[38] Philippon D., Godinho V., Nagy P.M., Delplancke-Ogletree M.P., Fernández A. (2012). Endurance of TiAlSiN coatings: Effect of Si and bias on wear and adhesion. Wear.

[39] Ren L.Q. (2011). Soil Adhesion Mechanics.

[40] Wang H.B., Wan Q., Zhou M., Xu G., Du X., Wei M., Meng L., Li S.J. (2020). Reduction of friction and soil adhesion of medium carbon steel via hard coating and surface texture. Coatings.

[41] Yang J., Liu Z., Cheng Q., Liu X., Deng T. (2019). The effect of wear on the frictional vibration suppression of water-lubricated rubber slat with/without surface texture. Wear.

[42] Ren L.Q., Han Z.W., Li J.Q., Tong J. (2006). Experimental investigation of bionic rough curved soil cutting blade surface to reduce soil adhesion and friction. Soil. Tillage Res..

